# Albumin coatings for counteracting uremic metabolites effects on host responses to biomaterials

**DOI:** 10.3389/fbioe.2025.1704352

**Published:** 2025-11-11

**Authors:** Indu Sharma, Mehdi Ghaffari Sharaf, Aishwarya Pawar, Ethan Lockwood, Larry D. Unsworth

**Affiliations:** Department of Chemical and Material Engineering, University of Alberta, Edmonton, AB, Canada

**Keywords:** iron oxide nanoparticles, BSA, uremic metabolites, chronic kidney disease, plasma proteins

## Abstract

Chronic kidney disease is a progressive condition characterized by a decline in kidney function that is the impetus for an increased retention of uremic metabolites (UMs) in the blood compartment which is correlated with adverse patient outcomes. The inefficient clearance of some UMs using membrane hemodialysis is a significant problem, and adsorptive materials are actively being researched to overcome this issue. Albumin is an abundant serum protein known to bind UMs and minimize non-specific protein adsorption at albumin-modified surfaces: two important aspects for designing modified surfaces for clearing UMs from blood. Herein, we anchored albumin to nanoparticles to understand if UM effects on protein-driven host responses to nanoparticles could be minimized. UM doped platelet-poor plasma was used to characterize protein-initiated clotting kinetics and immunoblot analysis of important protein mediators of the immune, fibrinolytic, and coagulation cascades. The amount of particles and density of adsorbed BSA could return clot formation to that seen for uremic plasma controls, but were unable to return these conditions to that seen for normal plasma. These surfaces significantly lower amounts of adsorbed C3, α_1_-antrypsin, Protein S, cleavage fragments of fibrinogen, prothrombin, factors XI and XII, and antithrombin compared to bare controls with UMs. However, in almost all cases the effect of UMs still led to dramatic increases in adsorbed proteins, and BSA films only reduced adsorption of IgG, vitronectin, prothrombin and antithrombin compared to normal plasma results. BSA films did ameliorate the effect of nanoparticles in uremic plasma. At these concentrations, these films were unable to adsorb enough UMs to negate their effects. This is an important milestone in the design of hemocompatible surfaces for clearing UMs from the blood.

## 1 Introduction

Chronic kidney disease (CKD) is the eighth leading cause of mortality worldwide, and ∼850 million people worldwide are affected (Antini et al., 2024; Liyanage et al., 2022; Francis et al., 2024). Currently, ∼2.5 million people receive renal replacement therapy, projected to increase to 5.4 million by 2030 (Liyanage et al., 2022; Bikbov et al., 2020). In the United States, treating CKD costs an estimated $48 billion annually while covering less than 1% of the Medicare population. China’s economy is projected to lose approximately $558 billion over the next decade due to the effect of CKD (Damien et al., 2016). Hemodialysis (HD) provides an immediate life-saving treatment but, despite intensive research and high treatment costs per patient, an inability to effectively clear large, middle or small protein-bound uremic metabolites (PBUMs) persists with poor patient outcomes: an unchanging average life span on HD of ∼3 years, an abysmal quality of life, and increased co-morbidities (Yang et al., 2021; Daneshamouz et al., 2021; Ma et al., 2020; Saito et al., 2000; Ragi et al., 2023). Adsorbent-based strategies are being sought to augment HD therapy and improve the clearance of uremic metabolites (UMs) (Ma et al., 2021; Rodrigues and Faria, 2023). Our group is one of the few to try to understand how uremic changes in the blood composition affect hemocompatibility of surfaces.

Magnetic nanoparticles (MNPs) have emerged as a novel class of advanced materials, demonstrating significant success in various medical applications (Malehmir et al., 2023; Montiel Schneider et al., 2022; Ojemaye and Okoh, 2019; Huang et al., 2024; Chehelgerdi et al., 2023). Iron oxide MNPs are uniquely suited as a platform for adsorbing UMs from blood due to their extensive surface area, numerous binding sites, tunable pore sizes and volumes, high biocompatibility and stability, easy functionalization, and separation capabilities facilitated by exogenous magnetic fields (Ojemaye and Okoh, 2019; Li et al., 2024; Li et al., 2023). However, the complex interaction between blood proteins and these particles remains ill-defined despite the obvious implications on biodistribution, biofunction, efficacy, and safety (Malehmir et al., 2023; Mollahosseini et al., 2020; Siedlecki, 2018; Sobczynski et al., 2015). Moreover, the effect of the composition of uremic blood on the hemocompatibility of materials is unstudied, even though this is a major area of application.

Albumin is commonly used to modify the surface of nanoparticles to improve dispersion stability and biocompatibility, the latter through reducing non-specific protein adsorption and related host responses (Laurent et al., 2008). Bovine Serum Albumin-coated iron oxide nanoparticles (BSA-MNPs) have shown potential in various biomedical applications, including as contrast agents in magnetic resonance imaging (MRI) and as carriers for targeted drug delivery (Hou et al., 2020; Elumalai et al., 2024). Moreover, albumin has been shown to bind multiple UMs that are otherwise hard to clear using HD, including some PBUMs and middle molecules (Rodrigues and Faria, 2023; Cheah et al., 2017). Recently, the interaction between common PBUMs (Carboxyl-Methyl-Propyl-Furan Propanoic Acid (CMPF), indoxyl sulfate (IS), p-cresyl sulfate (PCS), hippuric acid, phenylacetic acid, indole acetic acid) and albumin have been highlighted, (Watanabe et al., 2012; Dehghan Niestanak and Unsworth, 2023; Li et al., 2022). Although not fully defined for all UMs, PBUM-albumin interactions are thought to occur predominantly at Sudlow Sites I and II, and the retention of these sites is thought to be the mode of binding UMs to these modified particles (Daneshamouz et al., 2021; Ma et al., 2021; Dehghan Niestanak and Unsworth, 2023).

Previous work from our lab has detailed the dynamic adsorption of UMs to BSA-modified MNPs using a complex solution of 26 metabolites. Here, it was found that PBUMs adhered more effectively to albumin-modified nanoparticles than to unmodified controls, with adsorbed amounts independent of initial solution concentrations. Moreover, increasing incubation time showed that UM adsorption was dynamic, with changing amounts of adsorbed metabolites over time. To this end, we hypothesize that BSA-coated nanoparticles may adsorb UMs enough to reducing their effects on non-specific protein adsorption. Thus, BSA-coated MNPs were synthesized using a covalent binding technique to ensure a stable and uniform coating. Synthesized nanoparticles were characterized using transmission electron microscopy (TEM), zeta potential, and thermogravimetric analysis (TGA), to confirm size, morphology, dispersion stability, and surface chemistry. Plasma protein adsorption to BSA-coated MNPs was accomplished using plasma doped with a known and quantifiable set of 26 UMs (Table 1). Immunoblots were used to identify and qualify the adsorbed protein species, whereas the clotting kinetics were evaluated using recalcified plasma. All studies were conducted to understand the effect of the coating and the UMs on the protein adsorption and activation outcomes. This work advances our understanding of UMs’ impact on engineered surfaces’ hemocompatibility.

**TABLE 1:** Composition of uremic metabolite solution developed based on literature analysis of the blood of patients with kidney failure.

Uremic metabolite	Patient concentration (ave ±1 SD, mg/L)	Experimental concentration (mg/L)	Solution composition (mol%)	Ref
3-Deoxyglucosone	1.7 ± 1.0	1.7	0.131	Vanholder et al. (2003)
4-Ethylphenyl sulfate	0.242 ± 0.044	0.25	0.015	Itoh et al. (2012)
Argininic acid	<0.077	0.077	0.005	Vanholder et al. (2003)
Asymmetric dimethylarginine	0.385 ± 0.2884	0.385	0.024	Duranton et al. (2012)
Creatinine	136.0 ± 46.0	136	15.023	Vanholder et al. (2003)
Dimethyl glycine	0.5768	0.59	0.071	Vanholder et al. (2003)
Guanidinopropionic acid	0.288 ± 0.0183	0.29	0.028	Vanholder et al. (2003)
Hippuric acid	247.0 ± 112	236	16.454	Vanholder et al. (2003)
Homocysteine	8.1 ± 1.6	8.1	0.749	Vanholder et al. (2003)
Hypoxanthine	2.0 ± 1.6	2.0	0.184	Vanholder et al. (2003)
Indole acetic acid	2.03 ± 0.38	2.03	0.145	Duranton et al. (2012)
Indoxyl glucuronide	2.5 ± 0.3	2.5	0.101	Duranton et al. (2012)
Indoxyl sulfate	53.0 ± 91.5	53.0	3.106	Vanholder et al. (2003)
L-Asparagine	7.13 ± 3.7	7.13	0.674	Canepa et al. (2002)
L-Tyrosine	54.35 ± 16.3	54.35	3.747	Kopple et al. (1973)
p-Cresol sulfate	20.9 ± 12.2	20.9	1.387	Duranton et al. (2012)
Phenylalanine	8.92 ± 1.81	9.25	0.7	Wolfson et al. (1982)
p-hydroxy hippuric acid	4.43 ± 2.79	4.25	0.272	Schools et al. (1989)
Putrescine	0.00942 ± 0.00759	0.00942	0.01	Duranton et al. (2012)
Pyruvic acid	11.7 ± 8.6	11.7	1.661	Landon et al. (1962)
Spermidine	0.097 ± 0.045	0.096	0.008	Duranton et al. (2012)
Spermine	0.018 ± 0.0162	0.018	0.001	Vanholder et al. (2003)
Trimethylamine n-oxide	7.49 ± 2.39	7.5	1.248	Bain et al. (2006)
Uric acid	83 ± 13	83	6.169	Taki et al. (2007)
Uridine	9.8 ± 11.4	9.8	0.501	Vanholder et al. (2003)
Xanthosine	96.6 ± 62.9	96.6	4.247	Toshimitsu et al. (1998)

## 2 Materials and methods

### 2.1 Materials

Chemicals for synthesis: FeCl2·4H2O, FeCl3·6H2O, ammonium hydroxide solution (25%), Aminopropyltriethoxysilane (APTES, 99%), BSA (98%) purchased from Sigma Aldrich. Sodium hydroxide (Fisher Scientific), absolute ethanol (99.5%), phosphate-buffered saline (PBS) in HPLC-grade water (0.01M, pH 7.4, filtered, 0.22 µm) from Fisher Bioreagents and glutaraldehyde 70% EM grade from Electron Microscopy Sciences. Sodium phosphate dibasic heptahydrate, sodium phosphate monobasic monohydrate, and PBS tablets were also purchased from Fisher Scientific.

Chemicals for uremic metabolite solution: The following chemicals were obtained from Sigma-Aldrich: 3-Deoxyglucosone (≥75%), 3-Indoleacetic acid (≥98%), Asymmetric dimethylarginine (≥98%), Creatinine (≥98%), Dimethylglycine (≥99%), Hippuric acid (≥98%), Hypoxanthine (≥99%), Indoxyl glucuronide (≥98%), Indoxyl sulfate, Guanidinopropionic acid (≥97.5%), p-Cresol sulfate (≥95%), Phenylalanine (≥98%), Pyruvic acid (≥98%), Spermidine (≥99%), Spermine (≥97%), Trimethylamine N-oxide (≥95%), Uric acid (≥99%), Uridine (≥99%). Xanthosine and 4-Ethylphenyl sulfate (98%) were sourced from Apexbio (Houston, United States), and 4-Hydroxyhippuric acid (≥98%) was obtained from Cayman Chemical.

Materials used for immunoblot experiments: Platelet-poor humanparagraph plasma was obtained from Blood4Research program from Canadian Blood Services. Sodium dodecyl sulfate (SDS) and polyvinylidene fluoride (PVDF) membrane (Bio-Rad, Hercules, CA). TMB stabilized substrate (Promega, Madi-son, WI). BCA protein assay (Pierce™ BCA Protein Assay Kit, Thermo Fisher Scientific Inc.). For the full list of antibodies, see Supplementary Table S1).

### 2.2 BSA-MNP synthesis

Iron oxide magnetic nanoparticles were prepared following established protocols (Yallapu et al., 2011; Sharma et al., 2024). Briefly, 1 g of FeCl_2_·4H_2_O and 2.6 g of FeCl_3_·6H_2_O were dissolved in 25 mL of degassed water, stirred, and heated to 75 °C. After slowly adding 10 mL of 25% ammonium hydroxide and stirring, the reaction was terminated, followed by washing, magnetic separation, and vacuum drying. For BSA modification, 50 mg of nanoparticles were suspended in 5 mL of absolute ethanol, vortexed, and sonicated. After adding 35 µL of APTES under nitrogen and reacting overnight, nanoparticles were washed and resuspended in PBS with 0.6 mL of 70% glutaraldehyde. To apply the BSA coating, BSA was dissolved in half of the PBS volume, combined with the nanoparticle suspension, and shaken for 2 h. Coated particles were washed and stored in PBS at 4 °C.

### 2.3 BSA-MNPs characterization

TGA characterization of the BSA film on MNPs was done using the Pyris 1 (Perkin Elmer, United States). Here, freeze-dried powders were characterized using a temperature ramp of 10 °C/min from 25 °C to 800 °C, under ultra highly pure -N_2_. TEM micrographs were captured using a JEM-ARM200CF S/TEM (JEOL, Houston, TX, United States) at an accelerating voltage of 200 kV, and image analysis for MNP and BSA-MNP particle size determination was conducted using ImageJ on 48 nanoparticles. The effect of BSA coating conditions on the zeta potential was determined using the Zetasizer Nano ZS (Malvern Instruments, Malvern, United Kingdom), following previously published protocols (Sharma et al., 2024).

### 2.4 Protein adsorption

Human plasma experiments followed the guidelines of the research ethics board approvals from Canadian Blood Services 2022-21 and the University of Alberta Pro00002363 and Pro00116764. A concentrated stock solution of UMs (Table 1) was made immediately before incorporating into platelet-poor plasma. Each chemical was dissolved in LC-MS grade water, and uric acid in powder form was added last. It was then freeze-dried and 1 mL of plasma introduced to each UM aliquot, thoroughly mixed, and left at room temperature for 30 min to ensure the UMs were completely dissolved and evenly distributed within the plasma solution. Protein adsorption from plasma was accomplished by incubating MNPs with platelet-poor plasma following established protocols (Bahniuk et al., 2020). Briefly, 0.25 mL solution of different magnetic nanoparticles (i.e., Bare MNPs, MNPs-APTES-GA-BSA-0.2, MNPs-APTES-GA-BSA-2) prepared in 10 mM PBS were added to mixture of 1 mL UM-plasma at 37 °C for 2 h, followed by incubation with fresh PBS to remove the loosely adsorbed proteins.

### 2.5 SDS-PAGE and immunoblotting

To evaluate and identify individual proteins eluted from those adsorbed to different types of MNPs for varied concentrations, sodium dodecyl sulfate-polyacrylamide gel electrophoresis (SDS-PAGE) and immunoblotting techniques were used. The protein profiles of the eluted samples were analyzed using as per previously established methods (Bahniuk et al., 2020; Bahniuk et al., 2012). In summary, equal volumes of eluted samples (100 µL) were run on gels and transferred to polyvinylidene difluoride membranes. These membranes were then cut vertically into 2-mm wide strips, each being utilized for immunoblotting with a different primary antibody (1:1,000 dilution). HRP-conjugated secondary antibodies with TMB substrate were used for visualization. To ensure comparability of intensities, a consistent 10-min color development process was employed for all immunoblots.

### 2.6 Plasma recalcification assay

Clot formation kinetics and plateaus were evaluated using a recalcification turbidimetric assay where 100 μL of premix solution of plasma and metabolites were mixed with 25 μL of different MNPs in a PBS solution. The plasma was initially treated with PBS (10 mM) for 30 min, followed by incubation with the MNPs before the assay commenced. To conduct the turbidity test, 100 μL of 0.025 M CaCl_2_ was dispensed into a 96-well plate. The absorbance at 405 nm was recorded every minute for an hour using a BioTek ELx808 plate reader. All experimental steps were performed at a controlled temperature of 37 °C, and the entire process was repeated independently three times to ensure reliability.

### 2.7 Statistical analysis

Statistical analysis was conducted by analyzing the zeta potential of MNPs after every modification step and quantification of immunoblot data from three independent experiments to assess the total adsorbed amount for all types of MNPs (bare and BSA-modified particles). Statistical analysis was conducted using an ANOVA two way, considering unequal variances between the samples. Calculated p-values were defined as p < 0.01 and p < 0.05. No significant differences are labeled as “ns”.

## 3 Results and discussion

### 3.1 Particle size and film thickness

**FIGURE 1:**
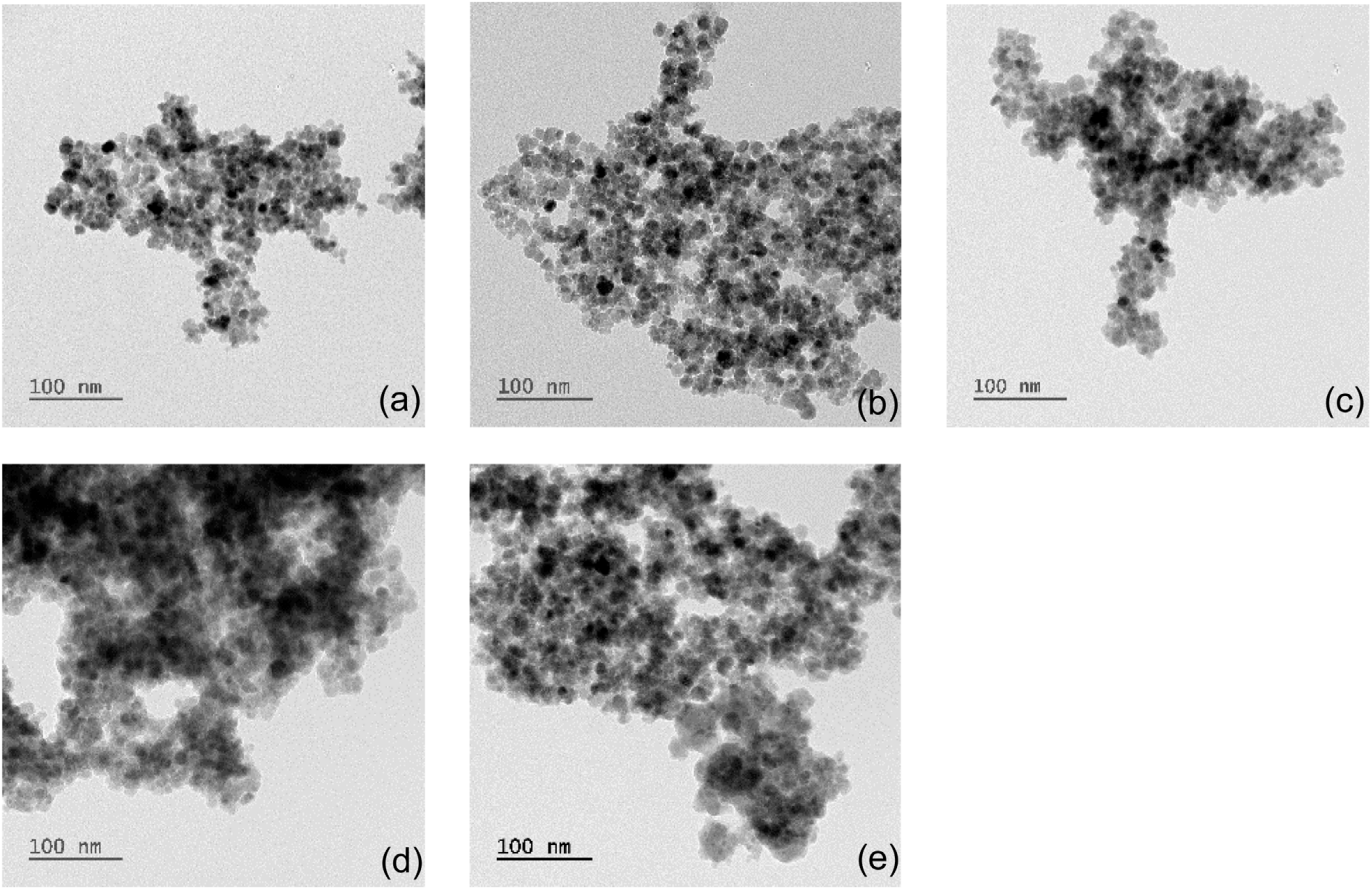
TEM images of **(a)** MNPs, **(b)** MNPs-APTES, **(c)** MNPs-APTES-GA, **(d)** MNPs-APTES-GA-BSA-0.2 and **(e)** MNPs-APTES-GA-BSA-2.

Bare and albumin-coated MNP size, shape, and uniformity were evaluated (Figure 1). Bare MNPs (Figure 1a) showed a consistent spherical morphology, with an average diameter of 10.6 ± 4.2 nm and a range of 8–14 nm (Table 2). Modifications with APTES and glutaraldehyde had minimal impact on size or shape, aligning with previous studies (Figures 1b,c) (Kalidasan et al., 2016; Zhao et al., 2010). TEM imaging also revealed an albumin layer around the MNPs, evident as a lighter perimeter in Figures 1d,e. The average diameters of coated MNPs were 20.4 ± 4.2 nm at a BSA solution concentration of 0.2 mg/mL and 35.7 ± 7.1 nm at 2.0 mg/mL (Figures 1d,e). The 0.2 mg/mL concentration formed a thin layer consistent with BSA’s hydrodynamic radius, while the higher concentration led to particle sizes from 27 to 42 nm, in agreement with previous studies (Baki et al., 2021; Mikhaylova et al., 2004). BSA-MNPs displayed reduced responsiveness to magnetic fields compared to bare MNPs. Higher albumin concentrations at MNPs-BSA-2 produced more globular shapes and larger aggregates, with some residual protein appearing as light deposits in the images, though a crosslinked albumin layer was confirmed. Statistical analysis was conducted via t-test using Origin 2024 on the unmodified MNPs, MNPs-APTES-GA-BSA, at both 0.2 mg/mL and 2 mg/mL concentrations. The comparisons produced p-values of less than 0.0001 between the bare MNPs for the 0.2 mg/mL and 2 mg/mL formulations, and less than 0.001 when comparing the 0.2 mg/mL and 2 mg/mL groups, indicating significant differences.

**TABLE 2:** Particle size and zeta potential measurements for bare and BSA-modified MNPs. Zeta potential values used three independent repeats, whereas particle diameter was determined from 48 randomly chosen particles per sample. All data are shown as average ±1 SD.

MNP type	Zeta potential (mV)	Particle diameter (nm)
Bare	−24.8 ± 2.8	10.6 ± 4.2
APTES	−17.7 ± 1	12.8 ± 3.5
APTES-GA	−9.2 ± 2	13.1 ± 2.8
APTES-GA-BSA-0.2	−23.2 ± 3	20.4 ± 4.2
APTES-GA-BSA-2	−32.4 ± 3	35.7 ± 7.1

### 3.2 Film surface charge

Zeta potential measurements offer insights into the impact of surface modifications on the exposed surface charge, indicating long-term stability when values exceed approximately ± 30 mV (Abou Gabal et al., 2022). The observed zeta potentials for the bare, APTES, APTES-GA, APTES-GA-BSA-0.2, and APTES-GA-BSA-2 MNP (Table 2) align with previous studies (Sharma et al., 2024; Mazario et al., 2017; Go et al., 2015). These results verify the effective binding of APTES and glutaraldehyde through the noted shifts in surface charge (Mazario et al., 2017). The addition of albumin significantly lowered the zeta potential, reflecting the protein’s negative surface charge at the given pH of the solution. A higher concentration of albumin solution produced a more pronounced negative surface charge (Mazario et al., 2017; Chubarov, 2022).

### 3.3 Film hydration and weight loss

The effect of the aminopropyltriethoxy silane, glutaraldehyde, and BSA film on film hydration and the total concentration of BSA within the film was assessed using TGA (Table 3). Weight loss at temperatures below 100 °C was linked to the evaporation of bulk water, and loss between 100 °C and 200 °C was attributed to vicinal water (Li et al., 2024). The unmodified MNPs showed minimal weight loss upon heating, indicative of their stability within this temperature range and lower level of interaction with water compared to BSA-modified surfaces. Furthermore, successive chemical modifications increased weight loss percentages, signifying successful surface coating.

**TABLE 3:** Thermogravimetric analysis for bare and BSA-modified MNPs. All data are shown as average ±2 SD.

Weight loss based on temperature				
MNP type	10 °C	100–200 °C	200–400 °C	800 °C
Bare	1.52 ± 2	3.06 ± 2	7.69 ± 2	9.63 ± 2
Bare-APTES	4.77 ± 2	6.22 ± 2	11.61 ± 2	15.92 ± 2
Bare-APTES-GA	2.30 ± 2	4.06 ± 2	10.32 ± 2	22.61 ± 2
Bare-APTES-GA-BSA-0.2	2.88 ± 2	5.70 ± 2	19.38 ± 2	41.37 ± 2
Bare-APTES-GA-BSA-2	14.15 ± 2	16.12 ± 2	25.71 ± 2	42.81 ± 2

APTES-modified samples showed distinct characteristics, and adding glutaraldehyde further enhanced thermal stability. BSA decomposes between temperatures of 250 °C–400 °C (Gebregeorgis et al., 2013). A pronounced peak is visible in the region up to 110 °C, which is linked to an endothermic effect signaling the denaturation of BSA (Daneshamouz et al., 2021; Michnik et al., 2006; Csach et al., 2012). A substantial change is observed in the temperature range of 180 °C–500 °C, attributed to the breakdown of BSA, glutaraldehyde, and the complex of amine functional groups. It is suggested that a distinct shift in the rate of maximum decomposition, correlating with different concentrations of BSA, serves as evidence for the adsorption of BSA onto the magnetic nanoparticles (Csach et al., 2012). As the concentration of BSA increased from 0.2 to 2 mg/mL, the observed peak became more pronounced and shifted towards a slightly greater weight loss. The decomposition occurring between 200 °C and 380 °C is related to the breaking of C-O and C-C bonds. Thus, the greatest mass loss within this range was observed for the MNPs-APTES-GA-BSA-2 configuration, validating TEM findings showing a higher albumin presence within these layers.

### 3.4 Total adsorbed protein

**FIGURE 2:**
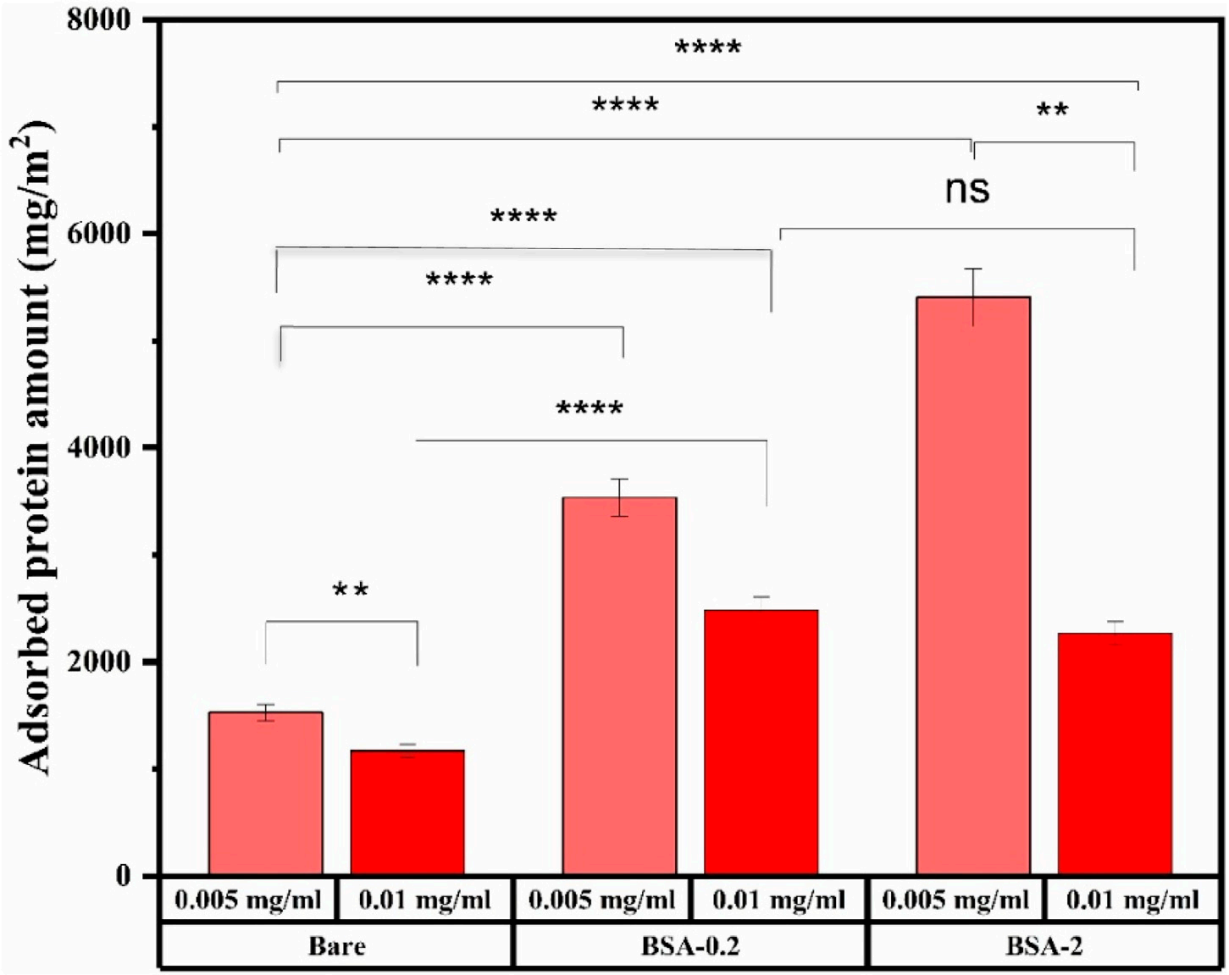
Representative results showing the total amount of UM-plasma protein adsorbed to each MNP system, determined using BCA assay. *Indicates p < 0.001, n. s represent not statistically significant, data represent ave ±1 SD, n ≥ 3. Statistical analysis was conducted using Two-way ANOVA where a p-value <0.05 showed statistical significance in protein adsorption amounts.

Complex dynamics are thought to be involved in protein adsorption in the presence of UMs, including protein-protein, metabolite-protein, and metabolite-protein-surface interactions. Metabolites may influence protein adsorption through mutual recruitment of metabolites and conformational changes in proteins induced by metabolites that may lead to changes in metabolite binding properties. This complex interaction space results in a dynamic protein corona surrounding nanomaterials, profoundly affecting their biological function (Chetwynd and Lynch, 2020). Introduction of UMs significantly affects protein adsorption, increasing it by 5–7 times across all nanoparticle types and concentrations, as illustrated by UM-plasma (Figure 2) and normal plasma results (Sharma et al., 2024). It was found that even though BSA was used to modify the bare MNP surface to inhibit protein adsorption, more protein adsorbed to BSA-MNPs relative to bare controls. Protein adsorption was also influenced by MNP concentration, where increased concentration yielded a notable decrease in adsorbed protein for all systems. These are counter-intuitive results. Adsorbed protein increased with films formed using 0.2 and 2 mg/mL, suggesting BSA may come off the surface and be measured as ‘adsorbed’ protein. If this were the case, increasing BSA-MNP concentration would doubly increase the adsorbed protein value, but the opposite was observed.

Moreover, the 0.01 mg/mL system showed a plateau in adsorption from 0.2 to 2 mg/mL BSA-MNP systems with similar amounts of adsorbed protein. It is not obvious why we see a decrease in adsorbed amount with increasing surface area of the system, except to say it is unrelated to the BSA film. It may reflect that the surface area of 0.01 mg/mL MNP concentration systems is so high it affects the saturation of the surface.

### 3.5 Clot formation

**FIGURE 3:**
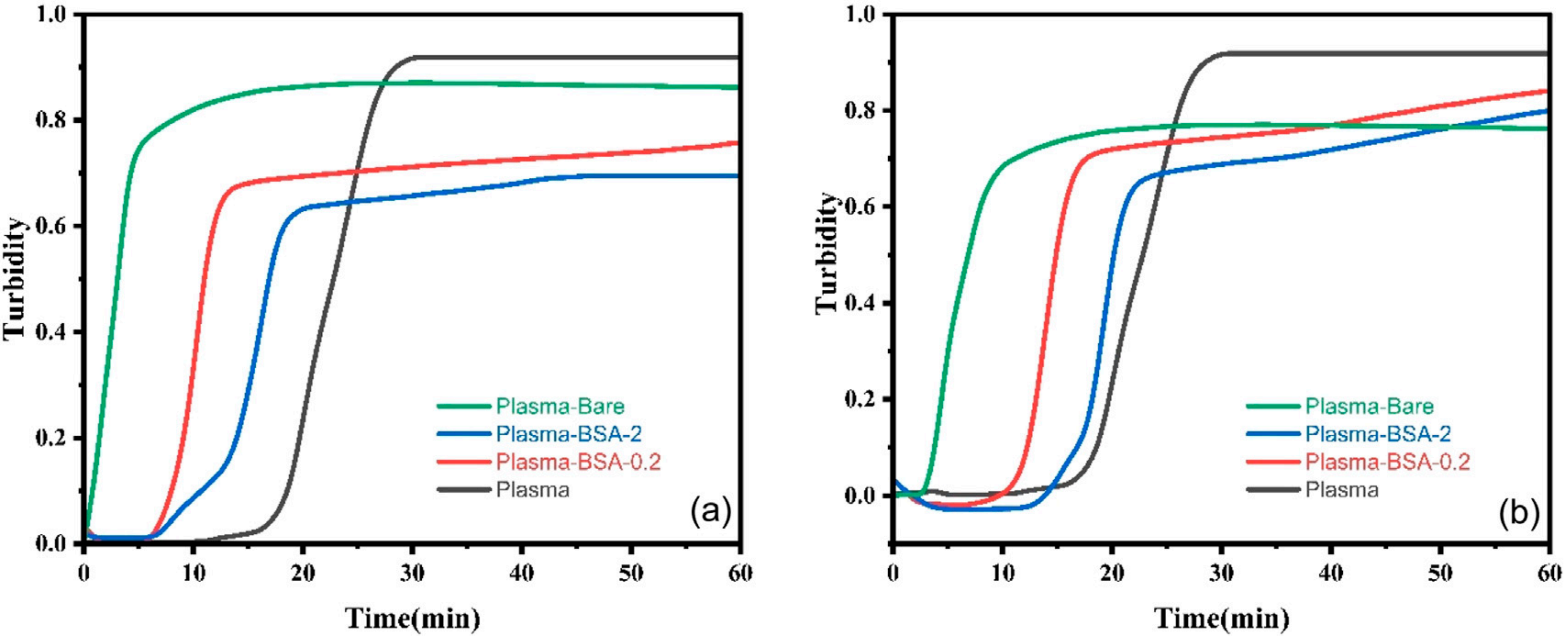
Representative plots of baseline-corrected average clot formation profiles in CKD platelet-poor human plasma over 60 min for Bare, BSA-0.2, and BSA-2 at **(a)** 0.005 mg/mL **(b)** 0.01 mg/mL MNP concentrations.

The interaction of Factor XII, high-molecular-weight kininogen, and prekallikrein with surfaces is well known to trigger the intrinsic activation of the contact pathway and induce clot formation (Bahniuk et al., 2020). Plasma clotting tests were conducted using unmodified and BSA-modified MNPs at 0.005 or 0.01 mg/mL particle solution concentration (Figures 3a,b).

MNP surface properties significantly influenced clotting properties. Without adding UMs, the bare MNPs accelerated the clotting process (6–7 vs. 18 min) and reduced clot density (i.e., turbidity, ∼0.7 vs. 0.85) and time to plateau (18–21 vs. 39 min) compared to plasma controls which performed as expected (Klok et al., 2020). Results for Bare MNPs were relatively insensitive to their solution concentration. Both BSA-MNP systems had clot onset times, densities, and plateau times between the bare MNP and plasma controls for both solution concentrations, illustrating the ability to reduce non-specific protein adsorption effects. However, both BSA-MNP system’s clot onset was drastically affected by the 0.005 or 0.01 mg/mL plasma concentration: BSA-0.2 with ∼9 vs. 17 min and BSA-2 with 6 vs. 14 min, respectively. The experiments were not designed to identify the mechanisms involved in the differences in clot onset as a function of BSA-MNP plasma concentration. Still, it does show that higher surface area available for the same amount of proteins lead to a more hemocompatible outcome.

Our previous work on CD-MPC-coated MNPs showed faster clot formation and plateau than BSA-coated particles (Li et al., 2023; Sharma et al., 2024). The clotting starting time for CD-MPC-coated MNP ranged from 1 to 2 min while for BSA coated MNPs 6–17 min. CD-MPC-coated MNP have plateau times of 5–6 min while for BSA coated MNPs depending on their concentration varied from 30 to 34 min. CD-MPC-coated MNPs tended to maintain or increase protein adsorption, possibly due to cyclodextrin’s ability to interact with protein structures. In contrast, BSA coatings often reduce adsorption, pointing to BSA’s role in shielding the nanoparticle surface from non-specific binding.

### 3.6 UM-plasma clotting

**FIGURE 4:**
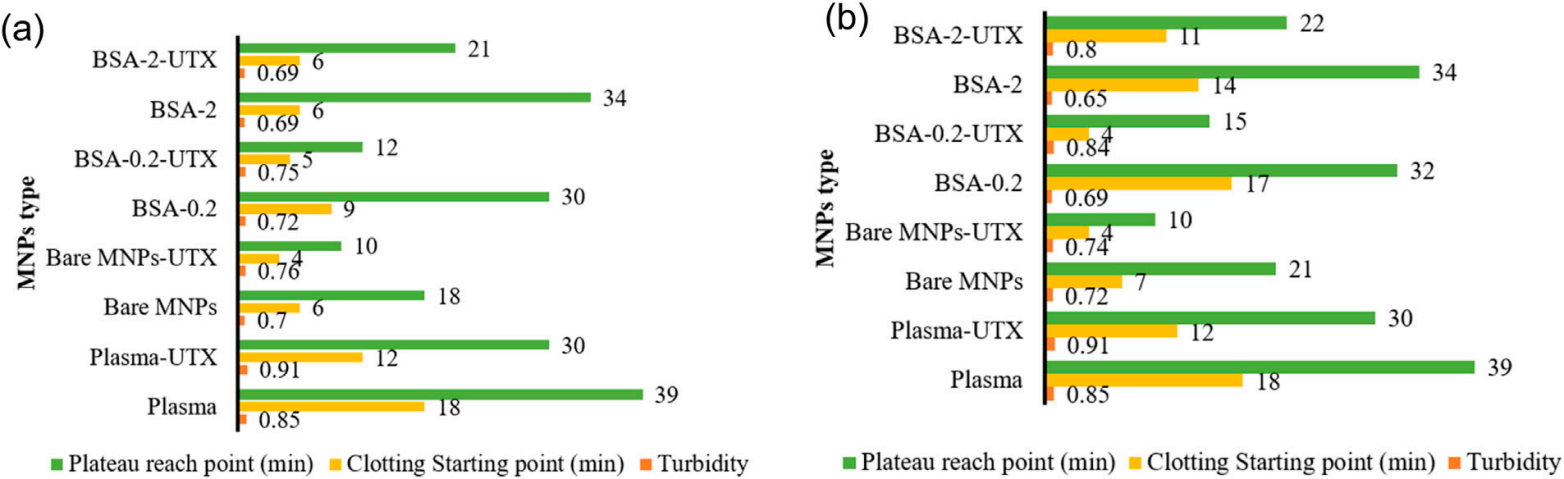
Representative plots of CKD patient vs. healthy plasma clotting starting point, plateau reach point and turbidity in platelet-poor human plasma over 60 min, with particles Bare, BSA-0.2, BSA-2 for **(a)** 0.005 mg/mL or **(b)** 0.01 mg/mL MNP concentrations.

The effect of UMs on the MNP clotting profiles (Figure 4) showed a faster onset of clot formation and plateau for all MNP types and concentrations, while clot density remained the same or increased. Bare MNPs, regardless of concentration studied, had clot and plateau onset times roughly halved in the presence of UMs. This effect was less pronounced with BSA-coated MNPs, showing intermediate to plasma and Bare-MNPs, which exhibit a slower approach to the clotting plateau. Dramatic differences were observed for BSA-0.2 systems at 0.01 mg/mL, where the clot onset time dropped from 17 to 4 min, the plateau time dropped from 32 to 15 min, and the density increased from 0.7 to 0.84 upon introducing the UMs. This suggests that the BSA-2 film may provide a more robust response against the influence of the UMs. Again, under the conditions studied, BSA films were unable to recover clotting parameters observed for normal plasma controls despite the fact some MNP conditions did overcome the influence the MNP surface had on clotting.

### 3.7 Adsorbed protein composition

Immunoblot bands were quantified using a 13-step grayscale system (Table 4; Supplementary Figure S1). The adsorption of different proteins and protein fragments, including albumin, immune-related proteins, and coagulation-related compounds, to various types of MNPs were evaluated at two MNP concentrations (0.005 and 0.01 mg/mL). The effect of UM addition on the protein content adsorbed to these surfaces was determined (Table 4), and the percent change relative to normal plasma was determined (Table 5). Our team has extensively discussed the role of each protein in plasma in previous publications (Li et al., 2023; Bahniuk et al., 2020).

**TABLE 4:** Relative intensities observed in the immunoblot of UM-incorporated plasma proteins adsorbed onto various MNP systems. The 0 and 12 indicate no band and maximum band intensity on the grayscale, respectively. The intermediate intensity values include 1–3 (very low band intensity), 4–5 (relatively low band intensity), 6–7 (moderate band intensity), 8–9 (relatively high band intensity), and 10–11 (high band intensity).

Plasma proteins			Fragment size (KD)	Bare		BSA-0.2		BSA-2		Legend
				0.005 (mg/mL)	0.01 (mg/mL)	0.005 (mg/mL)	0.01 (mg/mL)	0.005 (mg/mL)	0.01 (mg/mL)	
Immune response-related	Albumin		66	10	9	9	10	11	11	0
	C3	Whole C3	187	8	8	9	7	11	6	1–3
		α chain	115	9	11	10	9	6	9	4–5
		β chain	70	10	11	11	10	11	12	6–7
		Activation fragment	42	7	7	7	8	6	6	8–9
	IgG	Heavy chain	55	9	8	8	8	7	8	10–11
		Light chain	27	10	9	9	11	9	11	12
	Transferrin		77	12	11	12	12	11	11	
	Vitronectin		54	1	2	1	1	1	1	
	α1 antitrypsin		54	7	7	7	7	6	6	
	α2 macroglobulin		163	9	9	10	11	9	8	
	Protein S		75	1	2	1	2	0	1	
									
Coagulation related	Fibrinogen	α chain	68	9	8	10	11	10	8	
		β chain	56	9	8	10	10	8	8	
		γ chain	48	9	9	10	11	9	9	
		Cleavage fragments	<48	7	7	7	7	6	6	
	Prothrombin		72	4	5	4	5	3	3	
	Antithrombin		53	5	5	4	5	2	4	
	Factor XII		80	4	3	2	2	2	1	
	Factor XI		70	4	4	2	2	2	1	
	Prekallikrein		85	0	0	0	0	0	0	
	Protein C		62	0	0	0	0	0	0	
	Plasminogen		91	9	8	9	10	8	9	

**TABLE 5:** Percent change in the band intensities of protein adsorbed to Bare, BSA-0.2 and BSA-2 modified MNPs associated with UM addition.

Plasma Proteins			Fragment size (KD)	Bare	Bare	BSA-0.2	BSA-0.2	BSA-2	BSA-2
				0.005 (mg/mL)	0.01 (mg/mL)	0.005 (mg/mL)	0.01 (mg/mL)	0.005 (mg/mL)	0.01 (mg/mL)
**Immune response related**	Albumin		66	0	-25	0	0	22.2	37.5
	C3:	Whole C3	187	14.3	300	200	600	120	66.67
		α-Chain	115	0	57.14	11.11	12.5	-14.28	200
		β-chain	70	-9.09	0	10	0	22.22	50
		activation fragment	42	new band	new band	600	new band	500	new band
	IgG:	Heavy chain	55	-10	-20	14.28	14.28	-12.5	33.33
		light chain	27	0	-18.18	0	0	28.57	37.5
	Transferrin		77	9.09	0	9.09	9.09	10	10
	Vitronectin		54	new band	new band	-75	0	-50	0
	α_1_ antitrypsin		54	40	16.67	-30	-30	-40	-14.28
	α_2_ macroglobulin		163	800	350	233.33	1000	350	700
	Protein S		75	new band	new band	0	new band	-100	0
**Coagulation related**	Fibrinogen:	α chain	68	12.5	0	25	37.5	25	33.33
		β chain	56	0	-20	42.85	42.85	-11.12	60
		γ chain	48	28.57	12.5	42.85	57.14	28.57	200
		Cleavage fragments	<48	new band	new band	600	new band	500	new band
	Prothrombin		72	100	400	-20	-16.66	0	new band
	Antithrombin		53	150	66.66	-42.85	0	-66.66	new band
	Factor XII		80	new band	new band	new band	new band	new band	new band
	Factor XI		70	300	300	100	new band	100	0
	Prekallikrein		85	0	0	0	0	0	0
	Protein C		62	0	0	0	0	0	0
	Plasminogen		91	125	700	200	150	166.66	200

Color scale: 
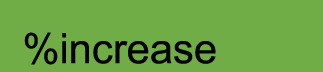
 
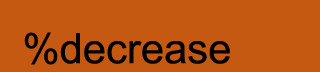
 
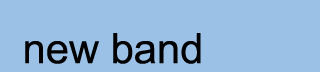

Albumin (66.5 kDa) adsorption can impact the adsorption of other plasma proteins (Klok et al., 2020). Small UMs, typically composed of an aromatic group with a polar component, can be bound by albumin, disrupting its transport function and potentially altering albumin’s structure (Yu et al., 2017; Viaene et al., 2013; Stegmayr, 2015). Albumin has been observed to exhibit relatively high levels of adsorption to BSA nanoparticles coated with poly-L-lysine and poly (ethylene glycol) (Yogasundaram et al., 2012), as well as to MNPs coated with 2-(methacryloyloxy)ethyl phosphorylcholine (MPC), known for its gold-standard low-fouling film properties (Li et al., 2023). Normal plasma adsorption showed a high level of albumin to BSA-grafted-MNPs (∼10 intensity), which was similar to our observation for UM-plasma adsorption showing that regardless of MNP solution concentration and thus total surface area a high level of adsorbed albumin (∼10 intensity) throughout (Table 4). The effect of UM incorporation into the plasma (Table 4) showed higher concentrations of bare MNPs decreased in albumin signal, and albumin increased in a similar amount for both concentrations of the BSA-2 system and not for BSA-0.2 systems relative to the normal plasma control.

#### 3.7.1 Adsorbed protein composition: Immune response-related proteins

C3 is essential for initiating the complement activation. Upon complement activation, C3 cleaves into a 42-kDa fragment (Li et al., 2023; Yogasundaram et al., 2012). Whole C3 (187 kDa) has been previously found in the protein corona of β-cyclodextrin and MPC-coated MNPs (Li et al., 2023). Prior studies on dextran-coated superparamagnetic iron oxide nanoworms (hydrodynamic diameter ≈140 nm) have revealed that proteins adsorbed to the surface facilitate the assembly of the complement component, with C3 primarily forming covalent attachments to adsorbed proteins rather than to the dextran shell (Chen et al., 2017). Normal plasma results have shown whole C3 adsorbed at very low to low levels (1-5 intensity) across BSA-grafted MNP systems, where higher MNP concentrations lead to 2-point lower intensity values for both BSA systems. Also, Bare MNP systems dropped 5 points in intensity with increased MNP concentration. Here, significantly higher band intensities for C3 were observed upon incorporating UM for all systems (8–11 vs. 1–5). These changes are illustrated in Table 4, where BSA-modified surfaces showed significantly increased intensities compared to bare controls at 0.005 mg/mL concentrations; at 0.01 mg/mL concentration, this trend was reversed, and BSA-2 was the lowest adsorbed amount. BSA-0.2 systems showed the highest increase in adsorbed amount, even compared to BSA-2 systems; the effect of UM addition was muted for BSA-2 films. This increased C3 adsorption was among the highest for all characterized proteins in the presence of the UMs.

Both the α chain (115 kDa) and the β chain (70 kDa) of C3 were characterized, where the whole α chain indicates a lack of activation and should coincide with the C3 activation fragment (42 kDa). Compared to normal plasma results, α chain adsorption with UM increased for almost all systems studied (Table 4), except BSA-2 (0.005 mg/mL) with an intensity of 6. This observation correlated with C3 activation fragments, where all systems showed a substantial increase in band intensity from 6-8 to 0–1 for normal plasma. Several bands appear only when UMs are present. UMs lead to massive C3 activation, whereas BSA-2 systems have slightly less activation than the other systems. This is confirmed through the ratio of C3 to α chain present with UM, C3 went up significantly, but α chain did not, confirming that these are lost through activation, which coincides the significant increase in activation fragment. In general, the BSA-2 coating had lower adsorbed amounts of the α chain but increased amounts of β chain relative to both other systems. The amount of α and β chains of C3 seemed to change relative to MNP concentration upon the addition of UMs: a significant increase for BSA-2 (0.01 mg/mL) in both α and β chains.

IgG plays a key role in the classical complement pathway, triggering the activation of the classical complement pathway (Li et al., 2023). Compared to bare MNPs, MPC coating has been shown to reduce the surface adsorption of IgG (Li et al., 2023). BSA nanoparticle-coating with poly-L-lysine and poly (ethylene glycol) has been shown to inhibit IgG adsorption (Yogasundaram et al., 2012). No significant change in the overall adsorption patterns of the IgG light chain (27 kDa) or heavy chain (55 kDa) was observed for UM-plasma.

Transferrin (77 kDa) is a glycoprotein that mediates iron transport and hemostasis in the plasma (Bruhn and Spellberg, 2015). Previous immunoblot studies on MPC-coated MNPs have shown relatively high adsorption levels of transferrin (Li et al., 2023). Similarly, high adsorption levels of transferrin have been observed in poly (acrylic acid)-coated metal-oxide nanoparticles (Ortega et al., 2020). It was observed that Transferrin did increase marginally upon UM incorporation, however, it was already at high adsorbed amounts in normal plasma and may be related to the nanoparticle material.

Vitronectin is involved in the regulation of the complement system. MPC-coated MNPs have shown moderate levels of vitronectin adsorption (Li et al., 2023), while this protein has not been detected in poly-L-lysine and poly (ethylene glycol)-coated BSA nanoparticles (Yogasundaram et al., 2012). Very low to low intensity levels of vitronectin (1-4 intensity) were seen, with bare particles not showing a detectable band for this protein. UM incorporation lead to minimal vitronectin levels, with all MNP types showing an intensity of 1, except for bare particles at the concentration of 0.01 mg/mL, which showed an intensity of 2.

α_1_-Antitrypsin (54 kDa) is a serine protease inhibitor. Studies have shown that elastin-like polypeptide nanoparticles can adsorb high amounts of α_1_-antitrypsin (Bahniuk et al., 2020). In contrast, BSA nanoparticles coated with poly-L-lysine and poly (ethylene glycol)-coated have not shown any adsorption of this protein (Yogasundaram et al., 2012). Moreover, MPC-coated MNPs have been shown to reduce the adsorption levels of α_1_-antitrypsin compared to uncoated MNPs (Li et al., 2023). Normal plasma results showed that BSA-modified MNPs adsorbed almost twice the amount of α_1_-antitrypsin compared to uncoated MNPs. UM incorporation led to a 3-point drop in intensity for α_1_-antitrypsin adsorption to BSA-modified systems (∼10–7) except for BSA-2 (0.01 mg/mL). Conversely, bare MNP intensity increased, suggesting that BSA films were able to mediate this adsorption.

α_2_-Macroglobulin is a versatile protease inhibitor essential for regulating the immune response. Plasma concentration of α_2_-macroglobulin is increased in hemodialysis patients. While β_2_-macroglobulin is the major component of dialysis-related amyloidosis, complexes of circulating α_2_-macroglobulin and β_2_-macroglobulin have been detected in hemodialysis patients suggesting these complexes may play a role in dialysis-related amyloidosis (Lagrange et al., 2022; Argiles et al., 1993; Motomiya et al., 2003). α_2_-macroglobulin has not been detected in the protein corona of poly-L-lysine and poly (ethylene glycol)-coated BSA nanoparticles (Yogasundaram et al., 2012), β-cyclodextrin-coated MNPs, or MPC-coated MNPs (Li et al., 2023). α_2_-macroglobulin was one of the plasma proteins that exhibited substantially increased adsorption in metabolite-treated plasma compared to normal controls, going from intensities of 1-2 to 8–11; reflected in extreme increases in percent change shown in Table 4.

#### 3.7.2 Adsorbed protein composition: Coagulation-related plasma proteins

Fibrinogen (340 kDa) is a key protein involved in blood coagulation. Fibrinogen levels are elevated in hemodialysis patients, a risk factor for cardiovascular complications (Song et al., 1999; Goodship, 2003). Surface adsorption of large amounts of fibrinogen can indicate active coagulation. Previous studies have found all three polypeptide chains of fibrinogen (Aα, 68 kDa; Bβ, 56 kDa; and γ, 48 kDa), and fibrinogen fragments (<48 kDa), in high quantities adsorbed to BSA nanoparticles coated with poly-L-lysine and poly (ethylene glycol) (Yogasundaram et al., 2012). Compared to incubation in normal plasma, BSA-MNPs showed a slight increase in α, β, and γ chains intensity when incubated with metabolite-treated plasma. There was a substantial increase in the adsorption of fibrinogen fragments (<48 kDa), which were not detected in most MNP systems and appeared only as a faint band in BSA-MNPs at high MNP concentrations, increasing from 0 to 6–7. UMs are associated with fibrinogen fragmentation in patients with chronic kidney disease, which may indicate that this drastic increase occurred through oxidative stress or enzymatic cleavage (Sreedhara et al., 1996).

Prothrombin (72 kDa) is the inactive precursor to thrombin. Compared to normal plasma, BSA-0.2 MNP systems did not significantly alter prothrombin adsorption in UM-plasma. However, bare MNPs exhibited a noticeable increase in prothrombin adsorption in UM-plasma, with intensity values rising from a very low value (∼2 intensity) to an intensity of ∼4. In high concentrations of BSA-2 MNP (0.01 mg/mL), the intensity values of prothrombin remained unchanged. At a low MNP concentration (0.005 mg/mL), a faint band with low intensity was observed for this protein, which was not seen in normal plasma. Previous studies have shown that the prothrombin band was not found adsorbed to poly-L-lysine and poly (ethylene glycol)-coated BSA nanoparticles in normal plasma (Yogasundaram et al., 2012).

While thrombin (the active form of prothrombin) is involved in coagulation, antithrombin (58 kDa) plays a key role in natural anticoagulation pathways. In UM-plasma, all MNP systems exhibited low antithrombin adsorption (∼5 intensity), except BSA-2 at 0.005 mg/mL MNP concentration, which showed very low antithrombin levels (2 intensity). Antithrombin adsorption increased in UM-plasma experiments for bare MNP. At low MNP concentration (0.005 mg/mL), BSA-MNPs showed decreased adsorption in UM-plasma compared to normal plasma. At a higher MNP concentration (0.01 mg/mL), the adsorption of antithrombin remained unchanged in the BSA-0.2 system. In the BSA-2 system, a low-intensity band (4 intensity) appeared for antithrombin in UM-plasma, which was absent in normal plasma.

Among contact-phase coagulation proteins (i.e., Factor XI, Factor XII, plasma prekallikrein, and high-molecular-weight kininogen), neither prekallikrein nor kininogen showed any bands in either normal or UM-plasma across different MNP systems. Factor XI intensities increased upon metabolite addition, only bare MNPs showed a noticeable increase in Factor XI adsorption (∼4 intensity). While BSA MNPs showed a slight increase in Factor XI adsorption in the UM-plasma, this increase was negligible compared to that observed with bare MNPs. Factor XII was absent in all MNP systems in normal plasma. BSA MNPs showed a low-intensity band upon incubation with UM-plasma. Again, bare MNPs were the only systems with the highest increase (∼4) in Factor XII adsorption in UM-plasma.

In UM-plasma, a significant increase in plasminogen (8–10) intensity was observed in various MNP systems compared to normal plasma (1–4). However, the intensity values in UM-plasma reached high levels (∼9), with BSA-0.2 MNPs at 0.1 mg/mL concentration reaching a very high-intensity level (10). Surface adsorption of plasminogen is important because it is the primary zymogen in the clot lysis pathway, which binds to fibrin during fibrinolysis. Thus, it has been suggested that surface adsorption of plasminogen might lead to lysis of forming clots (Woodhouse and Brash, 1992).

Protein S amounts were similar to those observed in normal plasma across various MNP systems, showing either no band or only a faint band. No adsorption of Protein C was detected onto any MNP systems in UM-plasma, as observed with BSA MNPs incubated with normal plasma.

### 3.8 Effect of UM on adsorbed protein composition

For all types of MNPs, Factor XII, in particular, only appeared in the adsorption from UM systems, not healthy plasma. On introducing UMs and increasing MNP concentration, C3 α-chain and Prothrombin show significant increases in protein adsorption across all surfaces. Whole C3 showed an increase in band intensity for bare and BSA-0.2 MNP, whereas BSA-2 systems showed the opposite trend. This underscores the role of surface coatings in controlling protein adsorption. For C3 β-chain and plasminogen, a small gain was reported on bare and BSA-2 systems, while a decrease in BSA-0.2 MNPs was observed. Albumin, IgG Light chain, IgG heavy chain, α_1_-antitrypsin, and Fibrinogen β-chain showed reduced adsorption at higher concentrations for Bare MNPs, while for BSA-0.2, no change, and BSA-2 system incremental gain has been reported. Transferrin decreased in the adsorbed amount reported on bare MNPs, while there was no change for both types of BSA systems. A huge increase in protein adsorption intensity appeared for α_2_-macroglobulin, with increases in Fibrinogen α-chain and γ-chain Antithrombin for BSA MNPs and vice versa for bare MNPs; suggesting that higher MNP concentrations enhance protein-surface interactions. In healthy plasma, C3 activation fragment, Vitronectin, Protein S, and Fibrinogen cleavage fragments, was not detected on bare surfaces but appeared significantly on introduction of uremic metabolites, with increase on BSA systems indicating that UM may alter protein properties to facilitate adsorption. These observations suggest a robust interaction with various surface chemistries. Notably, in the presence of UM, maximum adsorption occurred on almost all types of surfaces. However, Prekallikrein and Protein C did not adsorb on any surfaces, even with UM present. This lack of adsorption across all conditions suggests that their molecular structures or surface interaction properties inhibit binding. Understanding these dynamics is essential for optimizing protein-surface interactions in biomedical applications, which can lead to improved performance of biomaterials.

### 3.9 Quantification of protein adsorption from healthy plasma: BSA-coated MNPs vs. CD-MNPs

A recent work investigating MNPs coated with poly (methacryloyloxy)ethyl phosphorylcholine-co-β-cyclodextrin (p (MPC-co-PMβCD)) film has revealed their effectiveness in adsorbing UMs, exhibiting both selectivity and dynamic interactions (Li et al., 2024). In contrast for healthy plasma, on comparing the p (MPC-co-PMβCD) film-coated MNPs with albumin-coated MNPs of differing surface coverages, the albumin-modified MNPs displayed enhanced hemocompatibility, demonstrated by extended clotting durations, reduced protein adsorption, and alterations in the proteome (Li et al., 2023; Sharma et al., 2024). On comparing the CD-MNPs and BSA-MNPs system for healthy plasma, distinct interaction patterns were observed based on the surface chemistry and the specific protein involved (Li et al., 2023; Sharma et al., 2024).

For BSA-coated MNPs, the adsorption of Albumin decreased with increasing concentrations of the BSA-MNPs system. In contrast, Albumin exhibited relatively high adsorption levels across all types of CD-MNPs, suggesting a strong interaction with the CD-modified surfaces. Fibrinogen demonstrated consistently high adsorption levels across all concentrations of the BSA-MNPs, indicating robust binding regardless of the presence of BSA. Furthermore, it also showed significant adsorption on CD-MNPs systems, particularly at higher CD ratios, which indicates its strong affinity for both surface types.

For IgG Heavy and Light Chains, there was a slight decrease in adsorption on BSA-modified surfaces compared to bare MNPs, while the CD-MNPs systems showed variable adsorption, generally lower at higher CD ratios. Transferrin, on the other hand, displayed high adsorption across both the BSA-MNPs and all CD-MNPs systems, indicating a strong affinity for the surfaces. Vitronectin showed minimal or no adsorption on the BSA-MNPs, suggesting effective blocking by BSA. Conversely, an increase in adsorption on CD-MNPs was observed with higher CD content, indicating selective interactions with this surface. Antithrombin exhibited moderate adsorption in the BSA-MNPs system, which slightly decreased at higher BSA concentrations, while for CD-MNPs systems adsorption was notably reduced, particularly at higher CD ratios. Prothrombin displayed a slight increase in adsorption from bare to BSA-0.2 surfaces, followed by a reduction on the BSA-2 surfaces, with low adsorption observed across all CD-MPC ratios. Thus, albumin modification of nanoparticle surfaces may provide a means of adsorbing UMs whilst preventing adverse host responses. However, the interactions of albumin-modified nanoparticles with uremic blood have not been well studied. This comparative analysis highlights the complex nature of protein-nanoparticle interactions and the crucial role of surface chemistry in protein adsorption dynamics. Such insights are vital for designing nanoparticles with desired biological identities and functionalities.

## 4 Conclusion

Uremic metabolites affect non-specific protein adsorption and subsequent protein-driven responses to biomaterials. Albumin is known to strongly bind medium and small uremic metabolites. For the first time, the effect of BSA coating on high surface area magnetic particles were evaluated to understand if they could adsorb enough uremic metabolites to ameliorate their effect on these host responses. Upon increasing the concentration of nanoparticles (both bare and modified), we noted a decrease in protein adsorption from UM-plasma. Also, a significant reduction in protein adsorbed amount occurred upon BSA modification: BSA-2 MNPs being the best. However, BSA films were unable to return the protein adsorption results to that seen for healthy plasma. Some BSA films recovered UM-plasma clotting results to near controls, minimizing the effect of the nanoparticle surface. However, these surfaces could not restore clotting properties to that seen for normal plasma controls. It was observed that factor XII, only appeared in the presence of UM. C3 α-chain and prothrombin significantly adsorbed, and transferrin significantly decreased. No adsorption was shown for prekallikrein and protein C. Albumin, IgG light chain, IgG heavy chain, α_1_-antitrypsin, fibrinogen β chain, C3 activation fragment, vitronectin, protein S, fibrinogen cleavage fragments, have mixed adsorption depending on surface type and concentration. In almost all cases the effect of UMs still led to dramatic increases in adsorbed proteins, and BSA films only reduced adsorption of IgG, vitronectin, prothrombin and antithrombin compared to normal plasma results. In the end, BSA films seemed to minimize the effect the nanoparticles had on various protein-driven host responses, but at these conditions could not remove enough UMs to negate their general effects. Such insights are vital for designing adsorbent systems that minimize protein-driven host responses whilst being able to bind UMs from plasma.

## Data Availability

The original contributions presented in the study are included in the article/Supplementary Material, further inquiries can be directed to the corresponding author.
