# Supplementary material for: Albumin coatings for counteracting uremic metabolites effects on host responses to biomaterials
Source: Front Bioeng Biotechnol. 2025 Nov 11;13:1704352. doi: 10.3389/fbioe.2025.1704352 (PMC12644085; doi:10.3389/fbioe.2025.1704352)
Supplement: Supplementary file 1 [file Table1.docx]

**Supplementary Information**

**Uremic Toxins Effects on Hemocompatibility of BSA-Coated Nanoparticles**

Indu Sharma, Mehdi G Sharaf, Aishwarya Pawar, and Larry D. Unsworth*

Department of Chemical and Material Engineering, University of Alberta, Canada, T6G 1H9

*Corresponding Author

Corresponding author address:

Larry D. Unsworth, Ph.D., P.Eng.

DICE 13-390

University of Alberta, Edmonton, AB T6G 2V4

Email: lunswort@ualberta.ca

Ph: 780-492-6020

Fax: 780-492-2881

| 1.  | 2.  |
| --- | --- |
| 3.  | 4.  |
| 5.  | 6. 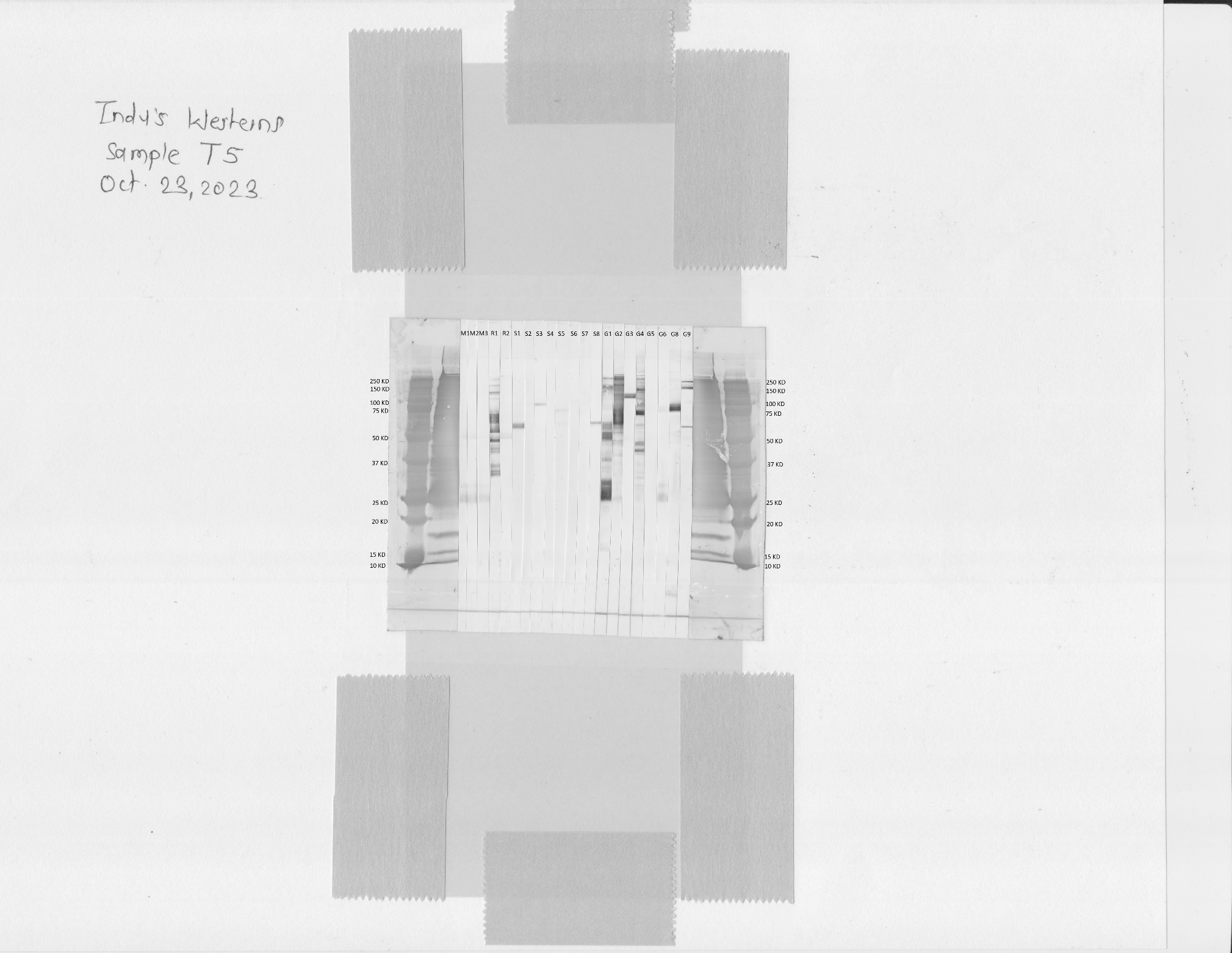 |

Figure S1. Representative reassembled western blot membrane of eluted plasma proteins from CKD patients plasma (1-2) bare MNPs, (3-4) Particle BSA(0.2), (5-6) Particle BSA(2). The middle strips (1-21) are processed in western blots, and two side pieces are non-specifically stained with colloidal gold stain.

| I | Ladder |
| --- | --- |
| II | Protein sample stained with non-specific colloidal gold |
| 1 | Ani-kininogen (light) |
| 2 | Ani-kininogen (heavy) |
| 3 | Anti-factor I |
| 4 | Anti-fibrinogen α, β, and γ |
| 5 | Anti-fibronectin |
| 6 | Anti-Alpha1 antitrypsin |
| 7 | Anti-thrombin |
| 8 | Anti-prothrombin |
| 9 | Anti-protein C |
| 10 | Anti-vitronectin |
| 11 | Anti-protein S |
| 12 | Anti-Prekallikrein |
| 13 | Anti-antithrombin |
| 14 | Anti-IgG |
| 15 | Anti-human albumin |
| 16 | Anti-plasminogen |
| 17 | Anti-C3 |
| 18 | Anti-factor XII |
| 19 | Anti-factor XI |
| 20 | Anti-transferrin |
| 21 | Anti-alpha2 macroglobulin |

Table S1. Primary antibodies against human plasma proteins used in immunoblot studies.

| **Anti-Human Plasma Antibody** | **Host** | **Vendor** |
| --- | --- | --- |
| Albumin | Goat | OEM Concepts, Saco, ME, USA |
| Antithrombin | Sheep | Cedarlane Laboratories, Hornby, Ontario, Canada |
| Complement factor 3 | Goat | Calbiochem, Gibbstown, NJ, USA |
| Factor I | Mouse | Invitrogen; Thermo Fisher Scientific Inc. |
| Factor XI | Goat | Cedarlane Laboratories, Hornby, Ontario, Canada |
| Factor XII | Goat | Cedarlane Laboratories, Hornby, Ontario, Canada |
| Fibrinogen | Rabbit | Calbiochem, Gibbstown, NJ, USA |
| Fibronectin | Rabbit | Cedarlane Laboratories, Hornby, Ontario, Canada |
| IgG | Goat | Sigma-Aldrich, St. Louis, MO, USA |
| Kininogen (heavy chain) | Mouse | US Biological, Swampscott, MA, USA |
| Kininogen (light chain) | Mouse | US Biological, Swampscott, MA, USA |
| Plasminogen | Goat | Cedarlane Laboratories, Hornby, Ontario, Canada |
| Prekallikrein | Sheep | Cedarlane Laboratories, Hornby, Ontario, Canada |
| Protein C | Sheep | Cedarlane Laboratories, Hornby, Ontario, Canada |
| Protein S | Sheep | Cedarlane Laboratories, Hornby, Ontario, Canada |
| Prothrombin | Sheep | Cedarlane Laboratories, Hornby, Ontario, Canada |
| Thrombin | Sheep | Cedarlane Laboratories, Hornby, Ontario, Canada |
| Transferrin | Goat | Sigma-Aldrich, St. Louis, MO, USA |
| Vitronectin | Sheep | Cedarlane Laboratories, Hornby, Ontario, Canada |
| α1-Antitrypsin | Sheep | Cedarlane Laboratories, Hornby, Ontario, Canada |
| α2-Macroglobulin | Goat | Sigma-Aldrich, St. Louis, MO, USA |
